# Temporal Changes in HbA1c Levels after Sacubitril/Valsartan Initiation in a Real-world Japanese Cohort: REVIEW-HF Registry

**DOI:** 10.31662/jmaj.2024-0242

**Published:** 2024-11-11

**Authors:** Yukihiro Watanabe, Yoshiaki Kubota, Takuya Nishino, Takahito Nasu, Shunsuke Ishii, Nobuyuki Kagiyama, Keisuke Kida, Wataru Fujimoto, Koshiro Kanaoka, Shingo Matsumoto

**Affiliations:** 1 Department of Cardiovascular Medicine, Nippon Medical School, Tokyo, Japan; 2 Department of Health Care Administration, Nippon Medical School, Tokyo, Japan; 3 Division of Cardiology, Department of Internal Medicine, Iwate Medical University, Iwate, Japan; 4 Department of Cardiovascular Medicine, Kitasato University School of Medicine, Kanagawa, Japan; 5 Department of Cardiovascular Biology and Medicine, Juntendo University Graduate School of Medicine, Tokyo, Japan; 6 Department of Digital Health and Telemedicine, R&D, Tokyo, Japan; 7 Department of Pharmacology, St. Marianna University School of Medicine, Kanagawa, Japan; 8 Department of Cardiology, Hyogo Prefectural Awaji Medicine Center, Hyogo, Japan; 9 Department of Medical and Health Information Management, National Cerebral and Cardiovascular Center, Osaka, Japan; 10 Department of Cardiovascular Medicine, Nara Medical University, Nara, Japan; 11 Division of Cardiovascular Medicine, Department of Internal Medicine, Toho University Faculty of Medicine, Tokyo, Japan; 12 British Heart Foundation Cardiovascular Research Centre, University of Glasgow, Glasgow, UK

**Keywords:** Sacubitril/valsartan, Heart failure, Diabetes, Glycemic control, Asia

## Introduction

Sacubitril/valsartan has been established as a key medication for patients with heart failure (HF). The PARADIGM-HF and PARAGON-HF trials revealed that sacubitril/valsartan has a pleiotropic effect, providing not only cardioprotection but also improved glycemic control ^(1), (2)^. The mechanism of action is thought to improve insulin secretion and sensitivity by increasing natriuretic peptide levels and reducing the degradation of glucagon-like peptide-1 (GLP-1) ^(2)^.

However, clinical data on the glycemic effects of sacubitril/valsartan in real-world patients are limited ^(3), (4), (5), (6), (7)^. Patients encountered in daily practice differ in characteristics and overall risk compared with clinical trial participants. Thus, real-world evidence is crucial to provide treatment effect estimates across a more diverse patient population. In addition, because the PARADIGM-HF and PARAGON-HF trials included only a small proportion of Asians (approximately 15%) ^(8), (9)^, the applicability of their findings to Asian populations is limited. Furthermore, although these trials evaluated long-term changes in HbA1c levels 12 months after sacubitril/valsartan initiation, short- to midterm changes in HbA1c levels remain unexamined. Early intensive glycemic control is important for reducing the lifetime risk of diabetes-related complications and mortality ^(10)^.

We therefore aimed to investigate temporal changes in HbA1c levels within 12 months after sacubitril/valsartan initiation in patients with HF using a nationwide Japanese registry.

## Methods

The Real-world evidence of Angiotensin Receptor-Neprilysin Inhibitor in Patients with Heart Failure (REVIEW-HF) registry is a multicenter, observational study conducted at 17 large-scale hospitals in Japan. This study was designed to assess the clinical features of patients with HF who were newly prescribed sacubitril/valsartan ^(11)^. Patients <20 years old were excluded from this study. The study protocol, including the use of an opt-out consent method, was approved by the ethics committee of Toho University Omori Medical Center (no. M21257) and the local ethics committees of all participating institutions.

In the current analysis, we evaluated changes in HbA1c levels at 1, 3, 6, and 12 months among patients who had baseline HbA1c data and continued sacubitril/valsartan treatment until 12-month followup. The evaluation at each time point was performed in the following range of periods: 1 month (21-39 days), 3 months (70-110 days), 6 months (150-210 days), and 12 months (310-420 days). The longitudinal changes in HbA1c levels were analyzed using a linear mixed model. In addition, subgroup analyses were performed using the following groups: sex (male or female), body mass index (BMI; <25 or ≥25 kg/m^2^), left ventricular ejection fraction (LVEF; <40% or ≥40%), daily dose of sacubitril/valsartan (≤200 or >200 mg/day), presence of diabetes, and use of sodium-glucose cotransporter-2 (SGLT2) inhibitors. A two-sided *P* value <0.05 was considered significant. Statistical analyses were performed using SPSS version 26 (IBM, Armonk, NY).

## Results

A total of 470 patients who met the inclusion criteria were analyzed. Table 1 presents the clinical characteristics. The mean age was 68 years, and 336 (72%) participants were men. The mean HbA1c level was 6.40%, and the prevalence of diabetes was 39%. The usage rate of SGLT2 inhibitors was 37%.

**Table 1.** Clinical Characteristics.

	All patients (n = 470)
Age (years)	67.8 ± 14.6
Male sex	336 (71.5)
BMI (kg/m^2^)	23.9 ± 4.6
HbA1c (%)	6.40 ± 0.99
eGFR (mL/min/1.73 m^2^)	50.0 ± 19.7
NT-proBNP (pg/mL)	1,844 (841-3,622)
LVEF (%)	38.8 ± 14.3
HFrEF (LVEF < 40%)	272 (57.9)
HFmrEF (LVEF 40%-49%)	103 (21.9)
HFpEF (LVEF ≥ 50%)	95 (20.2)
Medical history	
Diabetes mellitus	184 (39.1)
Hypertension	328 (69.8)
Dyslipidemia	268 (57.0)
Coronary artery disease	173 (36.8)
Treatments	
Beta-blockers	402 (85.5)
Mineral corticoid antagonists	317 (67.4)
Loop diuretics	364 (77.4)
SGLT2 inhibitors	175 (37.2)

Data are presented as mean ± standard deviation or median (interquartile range) for continuous measures and n (%) for categorical measures. BMI, body mass index; eGFR, estimated glomerular filtration rate; LVEF, left ventricular ejection fraction; HFrEF, heart failure with reduced ejection fraction; HFmrEF, heart failure with mildly reduced ejection fraction; HFpEF, heart failure with preserved ejection fraction; NT-proBNP, N-terminal pro B-type natriuretic peptide; SGLT2, sodium-glucose cotransporter-2

**Figure 1.**
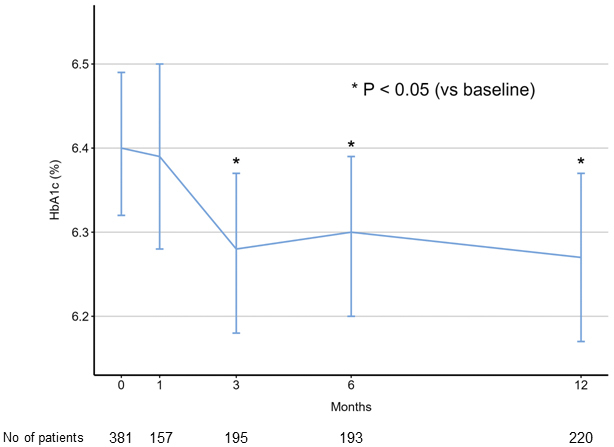
Temporal changes in HbA1c levels after sacubitril/valsartan initiation. HbA1c levels significantly decreased at 3, 6, and 12 months compared with baseline levels (*P* values for pairwise comparisons of estimated marginal means with Bonferroni adjustment). Error bars represent 95% confidence intervals.

**Figure 2.**
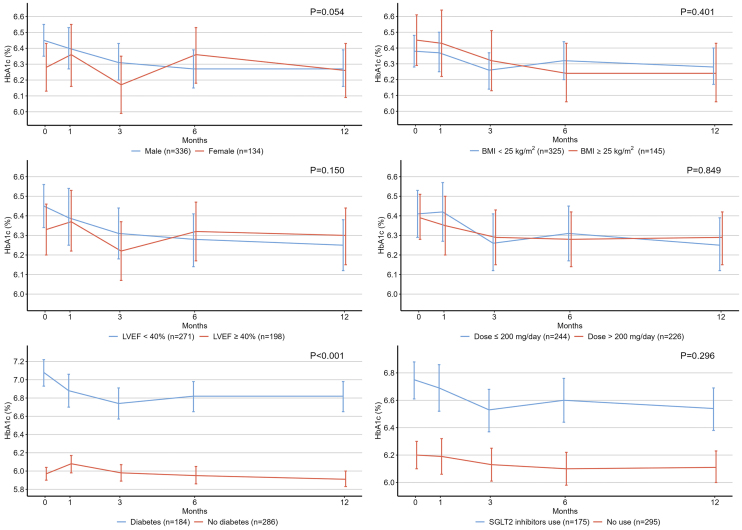
Subgroup analyses. Patients with diabetes showed a significantly greater decrease in HbA1c levels than those without diabetes. BMI, body mass index; LVEF, left ventricular ejection fraction; SGLT2, sodium-glucose cotransporter-2.

Figure 1 illustrates the temporal changes in HbA1c levels among the study patients. Compared with the baseline HbA1c level (6.40%), there was no significant change at 1 month (6.39%, *P* = 0.999), but a reduction in HbA1c levels was observed at 3 (6.28%, *P* = 0.012), 6 (6.30%, *P* = 0.048), and 12 (6.27%, *P* = 0.004) months (*P* values for pairwise comparisons of estimated marginal means with Bonferroni adjustment). Figure 2 shows the results of the subgroup analyses. Patients with diabetes showed a significantly greater decrease in HbA1c level than those without diabetes.

## Discussion

In this nationwide Japanese registry, we observed a significant decrease in HbA1c levels during the first 3 months following sacubitril/valsartan initiation. This decrease, compared with baseline HbA1c levels, was maintained for up to 12 months. The findings are consistent with those of previous landmark studies demonstrating a decrease in HbA1c levels after sacubitril/valsartan initiation ^(1), (2)^, supporting the potential benefit of this medication on glycemic control in patients with HF.

Studies examining the relationship between sacubitril/valsartan and glycemic control in real-word Asian populations are limited. One study in Japan demonstrated a significant decrease in mean HbA1c levels from 6.56% ± 0.68% to 6.49% ± 0.63% after a median followup period of 13 weeks in 150 patients prescribed sacubitril/valsartan for HF and/or hypertension ^(7)^. Another study in Korea found that patients treated with sacubitril/valsartan had lower HbA1c levels during followup compared with those treated with angiotensin-converting enzyme inhibitors or angiotensin II receptor blockers, although this difference was not significant after adjusting for confounders ^(6)^. These studies were conducted in single centers with relatively small sample sizes, whereas we provide findings from larger real-world data from 470 Japanese patients, indicating the potential benefit of sacubitril/valsartan in glycemic control.

Although long-term changes in HbA1c levels 12 months after sacubitril/valsartan initiation were evaluated in landmark trials ^(1), (2)^, we assessed short- to midterm changes in HbA1c levels and observed a significant decrease at 3 months. Studies on the short-term effects of sacubitril/valsartan on glucose metabolism are limited. In a single-center observational study involving patients with HF and/or hypertension, HbA1c levels modestly decreased from 6.56% ± 0.68% to 6.49% ± 0.63% over a median followup of 13 weeks ^(7)^. Another observational study showed improved insulin resistance at 3 months after sacubitril/valsartan initiation in patients with HF ^(12)^. Additionally, a randomized controlled trial involving obese patients with hypertension demonstrated that treatment with sacubitril/valsartan for 8 weeks, compared with amlodipine, improved insulin sensitivity ^(13)^. Thus, the beneficial effects of sacubitril/valsartan on glucose metabolism may become obvious early in the treatment process.

In the subgroup analyses, patients with diabetes had a significantly greater reduction in HbA1c levels than those without diabetes. This observation is consistent with the findings of the PARADIGM-HF and PARAGON-HF trials, indicating that the glucose-lowering effect of sacubitril/valsartan is modified by diabetes ^(2)^. Conversely, BMI and LVEF did not modify the effects of sacubitril/valsartan ^(2)^. Additionally, one observational study found no association between the dose of sacubitril/valsartan and the decrease in HbA1c levels ^(7)^. Our results are consistent with these findings, indicating that the glucose-lowering effect of sacubitril/valsartan may not be dose dependent. However, sacubitril/valsartan increases circulating GLP-1 levels in a dose-dependent manner ^(14)^. Further investigation is required to clarify the relationship between sacubitril/valsartan dose and its beneficial effects on glycemic control.

This study has some limitations that should be acknowledged. As an observational study, we cannot infer a causal relationship between sacubitril/valsartan use and glycemic control. Furthermore, this study was a single-arm analysis of patients treated with sacubitril/valsartan, making it impossible to compare the study with a control group that did not receive sacubitril/valsartan. Additionally, we do not have data on changes in medication during the followup period, and in particular, SGLT2 inhibitors may have affected HbA1c levels. Finally, a major limitation is the lack of information about antidiabetic medications, as the REVIEW-HF registry was not designed to evaluate the effects of sacubitril/valsartan on diabetes.

In conclusion, we observed a significant decrease in HbA1c levels during the first 3 months following sacubitril/valsartan initiation, with this decrease maintained for 12 months. These real-world data support the potential benefits of sacubitril/valsartan in glycemic control. Although the lowering effect of sacubitril/valsartan on HbA1c levels is clinically modest, it can be an effective therapeutic option for glycemic control.

## Article Information

### Conflicts of Interest

N.K. has received honoraria from Novartis Pharma, Boehringer Ingelheim Japan, Eli Lilly Japan K.K., and Otsuka Pharma and is affiliated with a department endowed by AMI Inc., Fukuda Denshi, KYOCERA, InterReha, and Philips Japan. K.K. has received honoraria from Otsuka Pharmaceutical Co., Ltd.; Novartis Pharmaceuticals Co., Ltd.; AstraZeneca K.K.; Ono Pharmaceutical Co., Ltd.; and Nippon Boehringer Ingelheim Co., Ltd. S.M. has received research grants and personal fees from Abott, Bayer Pharma, Boehringer Ingelheim, Daiichi-Sankyo, Medtronic, Novartis, Ono Pharma, Orbus Neich, Otsuka Pharma, and the Uehara Memorial Foundation. The remaining authors declare that there are no conflicts of interest.

### Acknowledgement

The authors would like to acknowledge all the investigators who contributed to the REVIEW-HF registry. We are also especially thankful to Drs. Atsushi Kikuchi (Osaka General Medical Center), Takeshi Ijichi (Tokai University School of Medicine), Tatsuhiro Shibata (Kurume University School of Medicine), Satomi Ishihara (Nara Medical University), Hideki Saito (Seirei Hamamatsu General Hospital), Riku Arai (Nihon University School of Medicine), Mirei Nabuchi (Teine Keijinkai Hospital), Kimitaka Nishizaki (Hirosaki University Graduate School of Medicine), Yoshiyuki Yazaki (Toho University Ohashi Medical Center), and Yu Horiuchi (Mitsui Memorial Hospital).

### Author Contributions

All authors contributed to the study conception and design. Yukihiro Watanabe, Takahito Nasu, Shunsuke Ishii, Nobuyuki Kagiyama, Keisuke Kida, Wataru Fujimoto, Koshiro Kanaoka, and Shingo Matsumoto conducted data collection. Yukihiro Watanabe, Takuya Nishino, Koshiro Kanaoka, and Shingo Matsumoto performed material preparation and analysis. Yukihiro Watanabe and Yoshiaki Kubota wrote the first draft of the manuscript. All authors critically revised the manuscript for important intellectual content and approved the final version. All authors are responsible for the interpretation of data and agree to be accountable for all aspects of the work, as well as in ensuring that questions related to the accuracy or integrity of any part of the work are appropriately investigated and resolved.

### Approval by Institutional Review Board (IRB)

The study protocol, including the use of an opt-out consent method, was approved by the ethics committee of Toho University Omori Medical Center (no. M21257) and the local ethics committees of all participating institutions.
